# Chemically Engineered *L. reuteri* Delivering αPD‐L1 and Gallium Ions via Metal‐Phenolic Networks Potentiate Anti‐Tumor Immunity and Ferroptosis

**DOI:** 10.1002/advs.202521638

**Published:** 2026-03-20

**Authors:** Tingting Zhang, Mengyi Xu, Yan Zhang, Zhiwen Li, Danqing Song, Jing Pang, Ke Li, Yanxiang Wang

**Affiliations:** ^1^ Institute of Medicinal Biotechnology Chinese Academy of Medical Sciences and Peking Union Medical College Beijing China; ^2^ Institute of Health and Medicine Hefei Comprehensive National Science Center Hefei Anhui China

**Keywords:** Bacterial‐based materials, ferroptosis, immune microenvironment, metal‐phenolic networks, synergistic tumor regression

## Abstract

Bacterial‐based materials, leveraging their inherent hypoxia and immunosuppressive microenvironment‐targeting capabilities, immunomodulatory effects, and drug delivery advantages, have emerged as promising strategies to modulate the tumor microenvironment (TME) for converting immunologically “cold” tumors into “hot” ones. This study innovatively functionalizes *Lactobacillus reuteri* (*L. reuteri*) using metal‐phenolic networks (MPNs) to co‐deliver αPD‐L1 antibodies and gallium ions (Ga^3^^+^) for synergistic anti‐tumor therapy. The MPN coating materials enable pH‐responsive drug release, whereby αPD‐L1 reverses immunosuppression and Ga^3^^+^ disrupts tumor iron metabolism, mimicking Fe^3^^+^ to induce selective ferroptosis. In vivo evaluations in B16 and LLC tumor‐bearing mouse models revealed that functionalized L. reuteri had the potential to reprogram the tumor immune microenvironment and significantly enhanced tumor regression. This system achieved multifunctional tumor regression in vivo, integrating bacterial hypoxia‐driven chemotaxis, MPN‐mediated ferroptosis, and immune checkpoint inhibitors (ICIs)‐driven immune activation. Thus, the multifunctional materials offer a novel strategy for treating refractory or immunologically “cold” tumors.

## Graphical abstract

A multifaceted chemical strategy, integrating bacterial tropism, metal‐ion interference therapy, and immunotherapy, resulted in significant tumor regression in murine models. A pioneering paradigm for the design of biohybrid materials was established, highlighting how sophisticated chemical engineering of living systems can unlock new avenues for combating refractory and immunologically “cold” solid tumors.

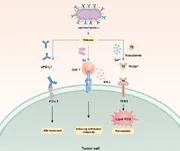

## 1 Introduction

Constructions of bacterial‐based bioactive materials have emerged as an innovative strategy that capitalizes on the unique biological properties of microorganisms and their dynamic interactions with the tumor microenvironment (TME) [1]. Solid malignancies frequently develop hypoxic niches due to chaotic angiogenesis and irregular vascular networks, creating oxygen‐deprived zones that serve as preferential colonization sites for both obligate anaerobes and facultative anaerobes [2]. The immunosuppressive TME, characterized by elevated lactate concentrations and acidic extracellular pH (typically ranging from 6.5 to 6.9), further creates a permissive ecosystem for the proliferation of certain bacteria [3]. Therefore, certain bacteria possess specific biological advantages for tumor treatment, including self‐propelled motility, hypoxia‐responsive chemotaxis, and tumor‐selective colonization capabilities. These features enable the delivery of engineered therapeutic payloads, thereby overcoming the diffusion limitations of conventional nanomedicines and macromolecular treatment [1, 4]. For example, attenuated *Salmonella* strains (e.g., VNP20009) demonstrate tumor‐selective colonization via auxotrophic mutations while maintaining immunogenic lipopolysaccharide profiles [5, 6]. *Clostridium sporogenes* has shown efficacy in lysing hypoxic tumor cores through localized prodrug activation of chemotherapeutic agents [7].

Recent studies indicate that *Lactobacillus reuteri* (*L. reuteri*), a commensal gut bacterium, modulates both local and systemic antitumor immunity. *L. reuteri* generates aryl hydrocarbon receptor ligands that enhance dendritic cell maturation while suppressing regulatory T cell activity [8]. Rational combination strategies integrating immune checkpoint inhibitors (ICIs) with conventional treatments such as cytotoxic chemotherapy, molecularly targeted agents, or epigenetic modulators have been clinically employed [9, 10, 11, 12]. Oral administration of *L. reuteri* synergizes with anti‐PD‐1/PD‐L1 therapies by increasing tumor‐infiltrating CD8^+^ T cell densities and reducing myeloid‐derived suppressor cell populations in colorectal cancer models [13]. This immunomodulatory synergy positions bacteria as promising candidates for overcoming ICI resistance in immunologically ‘cold’ tumors.

**FIGURE 1:**
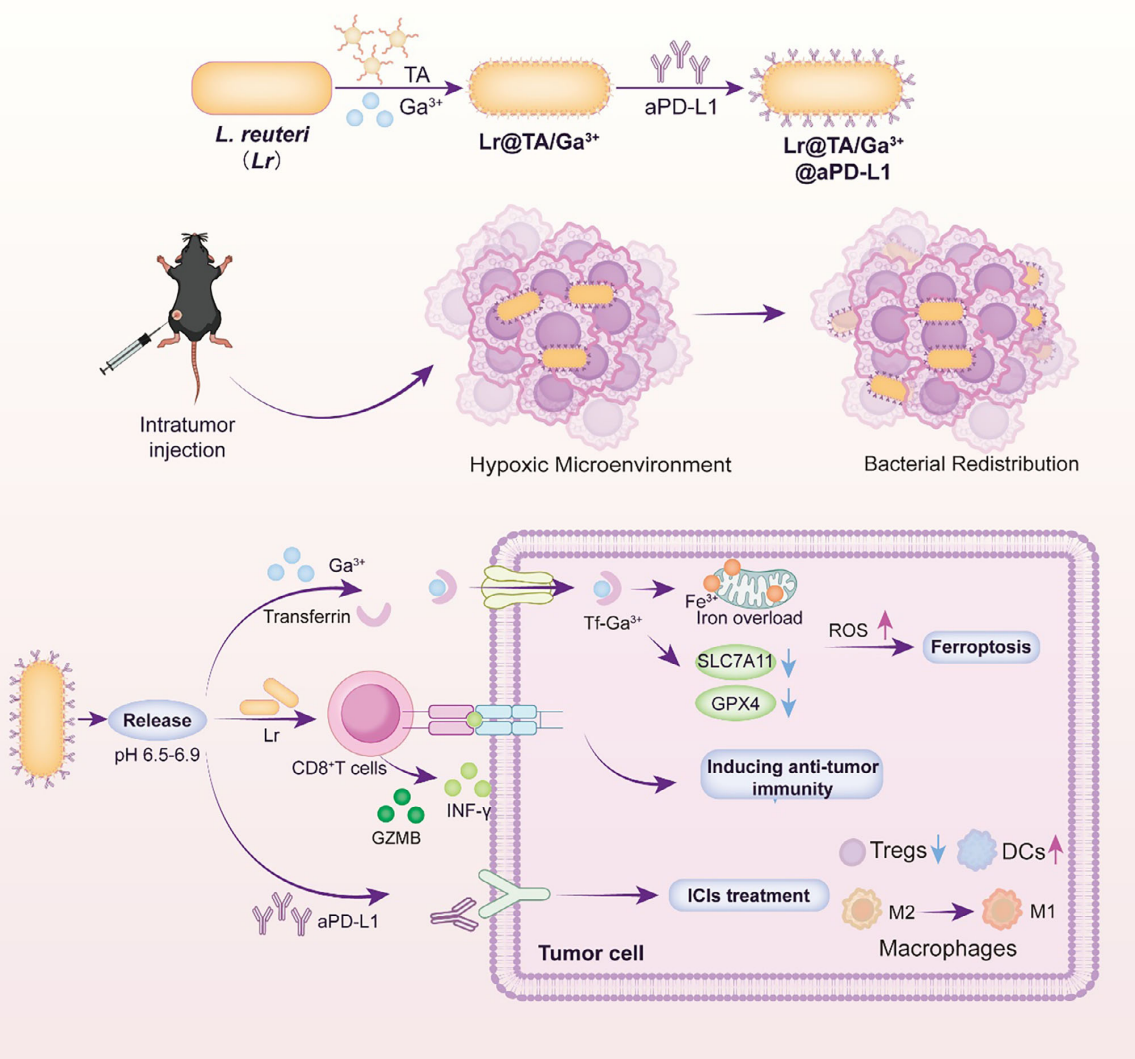
*L. reuteri* (*Lr*) was chemically modified to load a tannic acid (TA)‐gallium (Ga^3+^)‐based metal‐polyphenol network, on which PD‐L1 antibody was further immobilized. The hypoxic and slightly acidic tumor microenvironment favors the colonization of the engineered bacteria, followed by the dissociation of the metal‐phenolic network (MPN) to release PD‐L1 antibody, Ga^3+^, and *L. reuteri*. The immunogenicity of the bacteria activates CD8^+^ T cells to kill tumor cells; the PD‐L1 antibody functions as an immune checkpoint inhibitor; Ga^3+^ enters cells by competing for transferrin (Tf), inducing ferroptosis. The triple effects collectively lead to tumor cell death.

Genetically engineered bacteria have become a popular therapeutic approach, but they also carry potential risks of horizontal gene transfer and host mutation [14]. Building upon this reason, our study aims to develop chemically engineered bacterial carriers through surface functionalization to augment their therapeutic payload capacity, immune‐activating properties, and TME‐responsive behavior. As depicted in Figure 1, *L. reuteri* was functionalized using metal‐phenolic networks (MPNs) to co‐deliver αPD‐L1 antibodies and gallium ions (Ga^3^^+^). The modified materials facilitated: hypoxia‐driven tumor targeting; pH‐responsive drug release; reversal of immunosuppression through αPD‐L1 blockade; and disruption of iron metabolism to trigger tumor‐selective ferroptosis by Ga^3^^+^ imitation of Fe^3^^+^ when interacting with Transferrin (Tf). Thus, the chemically engineered bacteria achieved synergistic tumor regression. This bioactive material merges bacterial tropism, MPN‐mediated ferroptosis, and ICI‐promoted immune activation, offering a novel strategy to address the constraints of monotherapy and improve treatment of refractory or immunologically “cold” tumors.

## 2 Results

### 2.1 Design and Construction of MPN‐Functionalized *L. reuteri*

Tannic acid (TA), a naturally occurring polyphenolic compound derived from plant sources such as gallnuts and tea leaves, exhibits broad‐spectrum bioactivities, including antitumor, antimicrobial, and antioxidant effects. Its molecular structure, enriched with catechol and pyrogallol moieties, facilitates versatile molecular interactions through hydrogen bonding, π‐π stacking, hydrophobic forces, and metal coordination, rendering it a promising building block for supramolecular architectures [15, 16, 17]. These intrinsic properties, combined with its pH‐responsive behavior, biocompatibility, and biodegradability, have established TA‐based systems as prominent candidates for biomedical applications, particularly in targeted drug delivery, tissue regeneration, and antimicrobial coatings [16, 18]. For instance, TA has been employed to design pH‐sensitive nanocarriers for controlled chemotherapeutic release and to functionalize scaffolds for accelerated wound healing [19].

Leveraging the coordination ability of TA with metal ions, we designed a polyphenol‐metal network where TA coordinates with gallium ions to form a modified scaffold, creating a dissociable coating on bacterial surfaces. Due to its structural similarity to iron ions, gallium ions can compete with iron cofactors in metalloproteins, disrupting iron homeostasis and inducing cell death. Additionally, they can trigger ferroptosis through indirect pathways [20]. Building upon these advancements, we constructed a novel multi‐functional bacterial therapeutic system by integrating TA/Ga^3+^‐mediated MPNs with αPD‐L1 antibodies on the surface of *L. reuteri*—a probiotic strain previously proven to synergize with ICIs by modulating gut microbiota and enhancing antitumor immunity [13].

**FIGURE 2:**
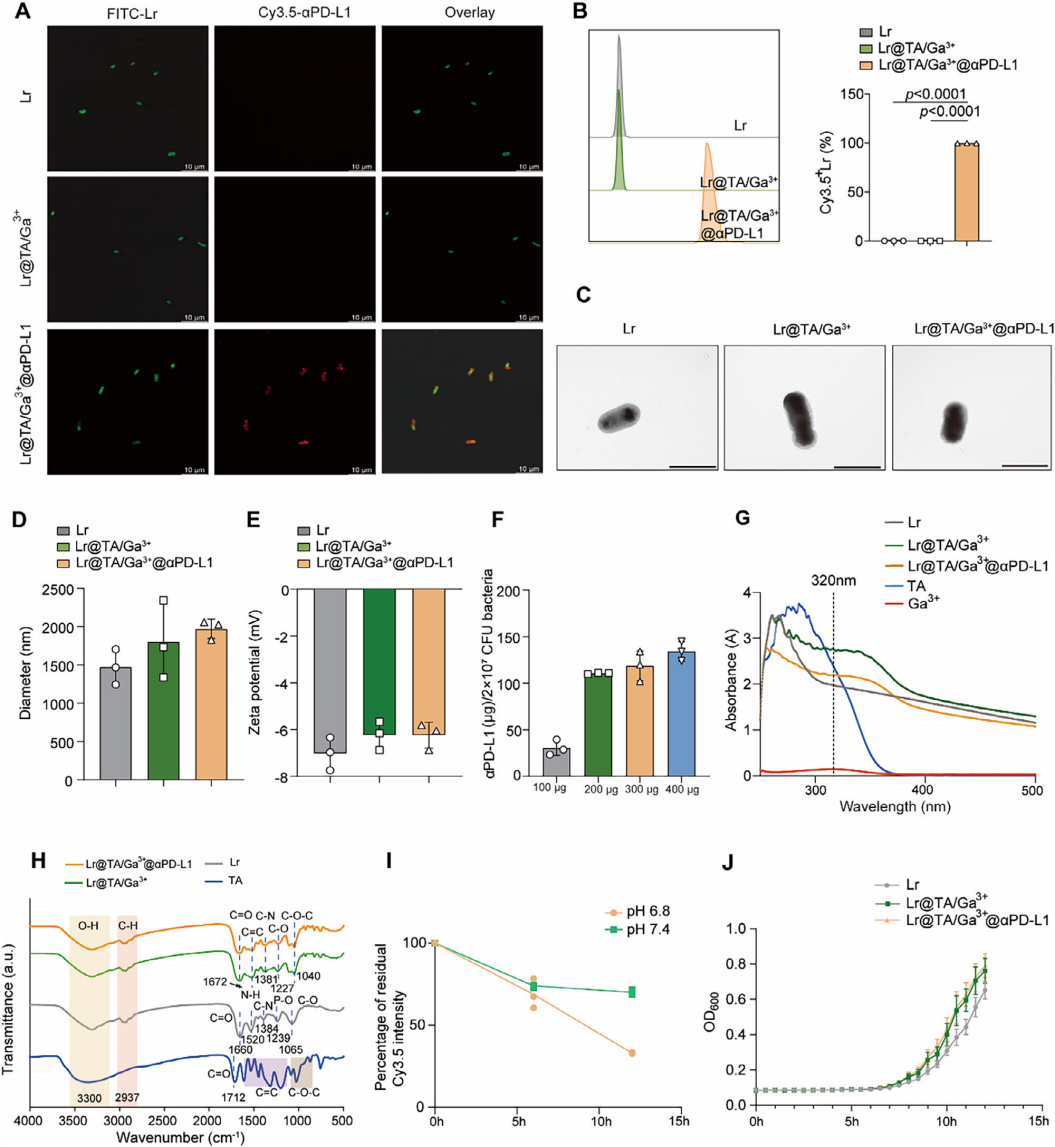
Structural characterization of modified materials. (A) Confocal imaging of modified materials. *Lr* and αPD‐L1 were labeled with FITC and Cy3.5, with excitation/emission wavelengths of 488/525 nm and 590/610 nm, respectively. Scale bar: 10 µm. (B) Flow cytometric analysis of Cy3.5 fluorescence intensity of *Lr*@TA/Ga^3+^@αPD‐L1 containing Cy3.5‐labeled αPD‐L1. (C)TEM images of *Lr*, *Lr*@TA/Ga^3+^and *Lr*@TA/Ga^3+^@αPD‐L1. Scale bar: 2 µm. Size (D) and Zeta potential (E) of *Lr*@TA/Ga^3+^@αPD‐L1. (F) Loading amount at different concentrations of αPD‐L1. UV absorption (G) and FTIR (H) of *Lr*, *Lr*@TA/Ga^3+^ and *Lr*@TA/Ga^3+^@αPD‐L1. (I) Release rate of *Lr*@TA/Ga^3+^@αPD‐L1 at pH 6.8 and 7.4, 37°C. (J) Growth curve of *Lr*, *Lr*@TA/Ga^3+^and *Lr*@TA/Ga^3+^@αPD‐L1. Statistical analysis was performed using one‐way analysis of variance (ANOVA) with the Tukey's post‐test (*n* = 3).

The fabrication process was conducted under mild, weakly alkaline conditions without compromising bacterial viability. A streamlined protocol was utilized: TA, Ga^3^^+^, and αPD‐L1 antibodies were successively co‐incubated with *L. reuteri* in Bicine buffer for 3 h under gentle agitation. This mild alkaline condition promoted TA self‐polymerization with Ga^3^^+^ coordination. To validate antibody conjugation, Cy3.5‐labeled αPD‐L1 antibodies were incorporated during the functionalization process, and the modified bacteria were analyzed by laser scanning confocal microscopy and flow cytometry, demonstrating robust fluorescence signals localized on bacterial surfaces, compared with unmodified *L. reuteri* (Figure 2A,B). Structural characterization via transmission electron microscopy (TEM) revealed a uniform, nanoscale TA‐derived coating enveloping the bacterial surface, contrasting with the unmodified controls (Figure 2C). Dynamic light scattering (DLS) measurements indicated a significant increase in hydrodynamic diameter from 1472.67 ± 230.04 nm (native *L. reuteri*) to 1970.33 ± 125.03 nm (*Lr*@TA/Ga^3+^@αPD‐L1) post‐modification, consistent with the formation of a TA‐Ga^3^^+^ composite layer group (1803.67 ± 506.92 nm, Figure 2D). As shown in Figure 2E, zeta potential of modified bacteria, including *Lr*@TA/Ga^3+^ (−6.24 ± 0.56 mV) and *Lr*@TA/Ga^3+^@αPD‐L1 (−6.23 ± 0.61 mV), exhibited significant shift compared to native *L. reuteri* (−7.02 ± 0.70 mV). Among the four modified groups treated with different concentrations of αPD‐L1, quantitative analysis via bicinchoninic acid (BCA) assays revealed that a starting αPD‐L1 concentration of 200 µg/mL yielded the highest loading efficiency of 54%, which means the antibody‐loading capacity is 108 µg αPD‐L1 per 2 × 10^7^ CFU bacteria (Figure 2F), ensuring sufficient immune checkpoint blockade efficacy. Inductively coupled plasma mass spectrometry (ICP‐MS) analysis revealed a gallium loading efficiency of 98.2%, demonstrating TA's chelating capacity for metallic gallium. Ultraviolet visible (UV–vis) spectroscopy further confirmed the successful incorporation of Ga^3^^+^, with a characteristic absorption peak at ∼320 nm corresponding to metal‐phenolate charge‐transfer complexes (Figure 2G). Infrared spectroscopy was used to analyze the interaction mechanisms of coatings (Figure 2H). The attenuation of O─H and N─H stretching vibrations near 3300 cm^−^^1^ in *Lr*@TA/Ga^3^^+^ and *Lr*@TA/Ga^3^^+^@αPD‐L1 results from the oxidation of TA's phenolic hydroxyl group to a quinone, which forms strong coordination bonds with Ga^3^^+^, significantly depleting free O‐H bonds. The presence of C═C vibrations at 1522 cm^−^^1^ confirms the successful incorporation of TA's aromatic ring structure into the composite system. The broadening and weakening of the peak originate from π‐π stacking interactions with surface proteins on *L. reuteri* and electronic effects induced by Ga^3^^+^ coordination, which restrict aromatic ring vibrations. In summary, the infrared spectroscopy results confirm that TA undergoes oxidation under alkaline conditions rather than simple physical adsorption. Its catechol/quinone structure forms stable coordination bonds with Ga^3^^+^, constructing a robust metal‐polyphenol network coating. Simultaneously, the TA/Ga^3^^+^ coating achieves tight bonding with the proteins on the Lactobacillus surface through interactions such as *π–π* stacking and hydrogen bonding.

Crucially, the TA‐Ga^3^^+^ network exhibited pH‐dependent release kinetics: under physiological conditions (pH 7.4, 37°C), approximately 30% of αPD‐L1 antibodies were released within 12 h, whereas 70% release occurred in tumor‐mimetic acidic environments (pH 6.8) within the same period (Figure 2I). This differential release profile ensures rapid immune activation in systemic circulation and drug delivery within the immunosuppressive TME. As shown in Figure 2J, the growth of the modified bacteria was not significantly affected compared with the bare bacteria, and the preserved bacterial viability ensured the therapeutic efficacy (Figure 2I). Viable counts also indicated that the modified coating did not affect bacterial viability (Figure S1A). Therefore, this multifunctional engineered system was designed to combine ICI‐driven immune reprogramming, *L. reuteri*‐driven autoimmune enhancement, and Ga^3^^+^‐induced ferroptosis, to address the limitations of conventional monotherapies.

### 2.2 Binding Capacity of Functionalized Materials In Vitro

**FIGURE 3:**
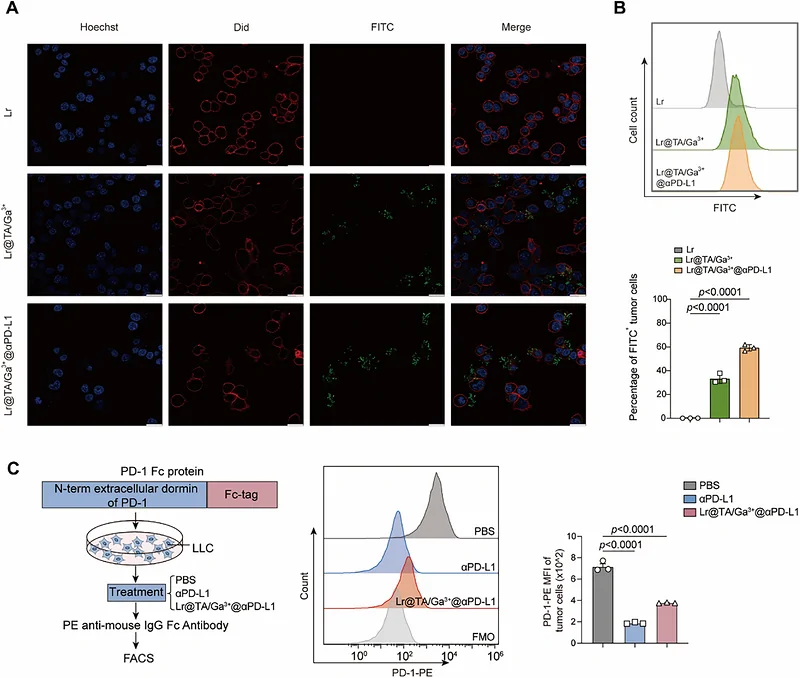
In vitro binding capacity of functionalized materials. (A) Confocal microscopy images showing co‐localization of LLC cells with *Lr*, *Lr*@TA/Ga^3+^ and *Lr*@TA/Ga^3+^@αPD‐L1, in which the bacteria were pre‐labeled with FITC. The cell nucleus was labeled with Hoechst, using excitation/emission wavelengths of 346/461 nm. The cell membrane was labeled with Did, using excitation/emission wavelengths of 644/665 nm. Scale bar: 10 µm. (B) Flow cytometric analysis of FITC fluorescence intensity in LLC tumor cells treated with *Lr*, *Lr*@TA/Ga^3+^, and *Lr*@TA/Ga^3+^@αPD‐L1 containing FITC‐labeled *Lr*. (C) The inhibition of PD‐1‐Fc binding to PD‐L1 on LLC tumor cells by PBS, free αPD‐L1, or Lr@TA/Ga^3^^+^@αPD‐L1 was assessed by flow cytometry. After pre‐treatment with PD‐1‐Fc, LLC cells were incubated with the indicated agents for 24 h and then stained with a PE‐anti‐mouse IgG Fc antibody. PD‐1/PD_L1 binding was quantified by the mean fluorescence intensity (MFI) of PD‐1 corresponding to PD‐1 attached to the tumor cells. Statistical analysis was performed using one‐way ANOVA with the Tukey's post‐test (*n* = 3).

The preservation of PD‐L1 antibody activity on functionalized bacteria is critical for antitumor immunity. To evaluate the binding efficacy of functionalized materials with tumor cells, we co‐cultured *Lr*@TA/Ga^3^^+^@αPD‐L1 with LLC cells in vitro. Confocal imaging revealed robust bacterial‐tumor cell binding, with functionalized bacteria exhibiting significantly enhanced adhesion compared to unmodified controls (Figure 3A). Flow cytometry analysis further confirmed the enhanced cell adhesion of functionalized bacteria (Figure 3B). Notably, bacteria modified solely with TA/Ga^3^^+^ also displayed improved tumor‐binding capacity, likely attributable to the adhesive properties of TA. To directly verify that the conjugation process did not compromise antibody activity, we performed a PD‐1/PD‐L1 blockade assay. The results confirmed that *Lr*@TA/Ga^3^^+^@αPD‐L1 effectively inhibited PD‐1/PD‐L1 interaction, retaining blocking activity comparable to that of free αPD‐L1 antibody (Figure 3C). Collectively, the functionalized bacteria demonstrate superior tumor‐targeting capabilities, highlighting their potential as promising candidates for antitumor immunotherapy.

### 2.3 Synergistic Therapy Efficacy of *Lr*@TA/Ga^3+^@αPD‐L1 In Vitro

**FIGURE 4:**
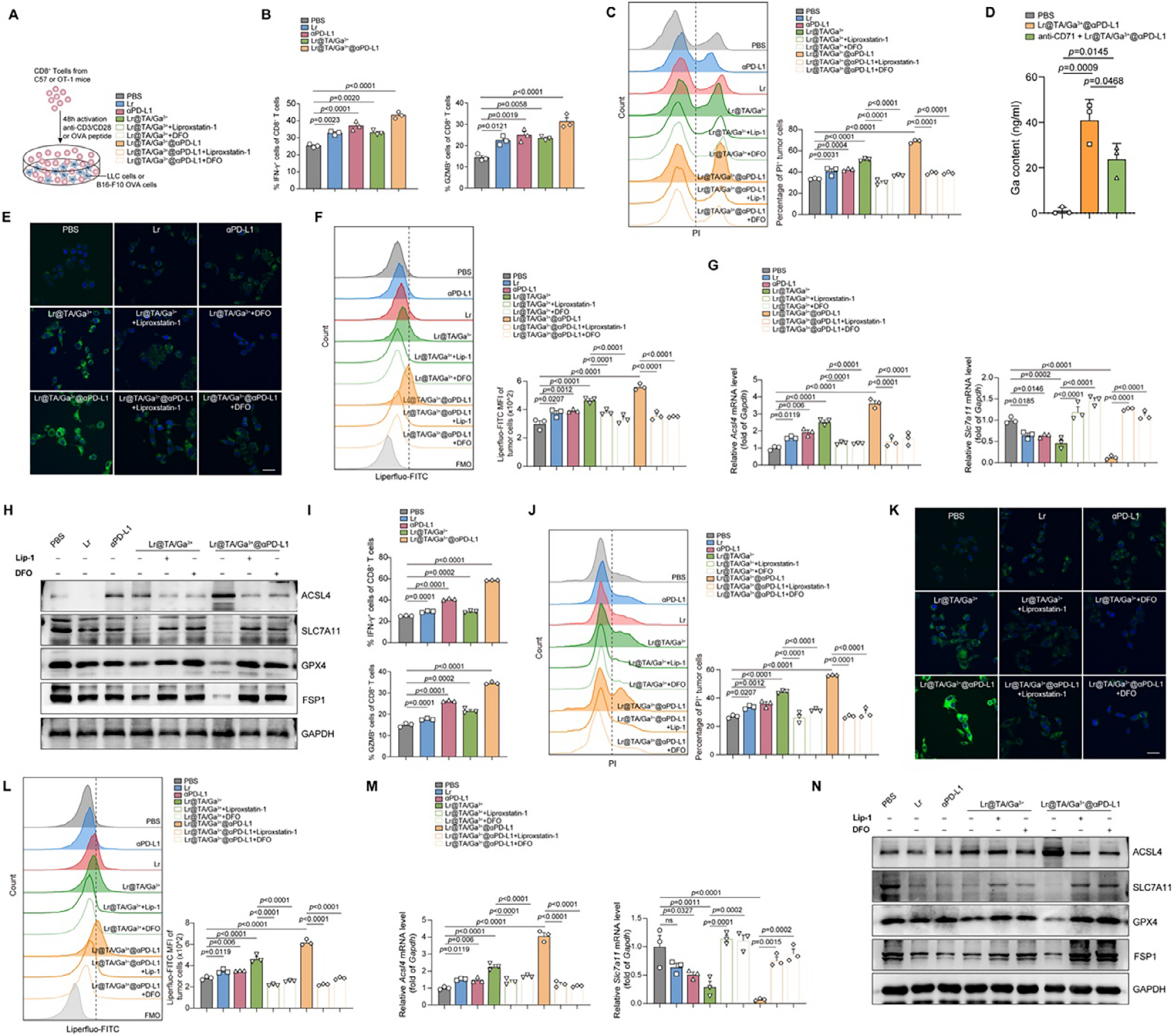
*Lr*@TA/Ga^3+^@αPD‐L1 enhanced the cytotoxicity of CD8^+^ T cells and promoted ferroptosis in tumor cells. (A) Flow chart showing the approaches of coculture tumor cells (LLC or B16‐F10 OVA) with mouse CD8^+^ T cells. CD8^+^ T cells isolated from C57BL/6 or OT‐1 mice were activated for 48 h and then cocultured with tumor cells for 36 h. (B) IFN‐γ and GZMB expression in CD8^+^ T cells cocultured with LLC cells after indicated treatments as determined by flow cytometry. (C) Flow cytometry histograms of PI^+^ in LLC cells cocultured with CD8^+^ T cells with indicated treatments. (D) Gallium uptake rate by LLC after treated with PBS or transferrin‐blocking antibodies (anti‐CD71). (E) Representative immunofluorescence images of the FerroOrange fluorescence intensity in LLC cells were measured using confocal microscopy. Scale bar: 10 µm. (F) Representative histograms of Liperfluo fluorescence intensity in LLC cells cocultured with CD8^+^ T cells following indicated treatments, measured by flow cytometer. (G and H) RT‐qPCR (G) and western blot (H) analysis of ACSL4, SLC7A11, GPX4, and FSP1 abundance in LLC with indicated treatments. (I) IFN‐γ and GZMB expression in CD8^+^ T cells from OT‐1 mice cocultured with B16‐F10 OVA cells after indicated treatments as determined by flow cytometry. (J) Flow cytometry histograms of PI^+^ in B16‐F10 OVA cells cocultured with CD8^+^ T cells from OT‐1 mice with indicated treatments. (K) Representative immunofluorescence images of the FerroOrange fluorescence intensity in B16‐F10 OVA cells were measured using confocal microscopy. Scale bar: 10 µm. (L) Representative histograms of Liperfluo fluorescence intensity in B16‐F10 OVA cells cocultured with CD8^+^ T cells from OT‐1 mice following indicated treatments, measured by flow cytometer. (M and N) RT‐qPCR (M) and western blot (N) analysis of ACSL4, SLC7A11, GPX4, and FSP1 abundance in B16‐F10 OVA with indicated treatments. Statistical analysis was performed using one‐way ANOVA with the Tukey's post‐test (*n* = 3).

We further examined the regulatory effect of *Lr*@TA/Ga^3+^@αPD‐L1 on the antitumor effects of CD8^+^ T cells. CD8^+^ T cells were activated and co‐cultured with tumor cells for 48 h (Figure 4A). Flow cytometry analysis showed that *Lr*@TA/Ga^3+^@αPD‐L1 significantly enhanced the antitumor activity of the CD8^+^ T cells, as indicated by increased expression of interferon‐gamma (IFNγ) and granzyme B (GZMB) (Figure 4B). The rate of tumor cells death (Propidium Iodide (PI)^+^ tumor cells) induced by CD8^+^ T cells was remarkably up‐regulated after *Lr*@TA/Ga^3+^@αPD‐L1 treatment compared with all other groups (Figure 4C). Gallium ions have been reported to induce ferroptosis by interfering with iron metabolism or dysregulating cell redox homeostasis, which releases immunogenic signals that activate anti‐tumor immunity and immune response [21, 22]. These activated CD8^+^ T cells then secrete IFN‐γ that further promotes ferroptosis by enhancing iron accumulation and lipid peroxidation, thereby establishing a positive feedback loop [23]. To investigate cellular uptake of Ga^3+^ from materials, we co‐incubated the materials with cells for 24 h and subsequently measured Ga^3+^ levels in the cells via ICP‐MS. The results showed that the intracellular Ga^3+^ content had increased significantly (Figure 4D), and Ga^3+^ uptake by cells could be inhibited by transferrin‐specific antibodies (anti‐CD71).

To explore if ferroptosis and concomitant lipid peroxide accumulation contributed to tumor death mediated by CD8^+^ T cells, fluorescent probes (Liperfluo and FerroOrange) detecting intracellular lipid reactive oxygen species (ROS) and the level of divalent iron (Fe^2+^) were cocultured with tumor cells, CD8^+^ T cells, and *Lr*@TA/Ga^3+^@αPD‐L1. LLC cells showed increased fluorescence intensity of FerroOrange and Liperfluo after *Lr*@TA/Ga^3+^@αPD‐L1 treatment (Figure 4E,F). In addition, prior to administering our therapeutic formulations, cells were pre‐treated with two ferroptosis inhibitors—liproxstatin‐1 (Lip‐1) and deferoxamine (DFO). The rescue experiments demonstrated that both Lip‐1 and DFO significantly reversed the promotion of tumor cell death (PI^+^) and the enhancement of lipid peroxidation and Fe^2+^ induced by both *Lr*@TA/Ga^3^^+^ and *Lr*@TA/Ga^3^^+^@αPD‐L1 in the presence of CD8^+^ T cells (Figure 4C,E,F). Furthermore, analysis of key ferroptosis markers at both transcriptional and protein levels revealed that, compared to other groups, treatment with *Lr*@TA/Ga^3^^+^@αPD‐L1 specifically upregulated the expression of the ferroptosis promoter ACSL4, while downregulating the expression of major ferroptosis suppressors, including GPX4, SLC7A11, and FSP1 (Figure 4G,H; Figure S2). Importantly, co‐treatment with Lip‐1 or DFO effectively inhibited the ACSL4 upregulation and restored the expression of GPX4, SLC7A11, and FSP1 in cells treated with *Lr*@TA/Ga^3^^+^ or *Lr*@TA/Ga^3^^+^@αPD‐L1 (Figure 4G,H; Figure S2). These data suggest that the cell death induced by *Lr*@TA/Ga^3^^+^@αPD‐L1 operates through a ferroptosis‐dependent pathway.

To further verify the unique characteristic of our platform in significantly enhancing antigen‐dependent adaptive immune responses, we performed an independent set of experiments using an antigen‐specific model. CD8^+^ T cells isolated from OT‐I transgenic mice were co‐cultured with B16‐OVA melanoma cells, which stably express the cognate ovalbumin antigen. Flow cytometry analysis showed that *Lr*@TA/Ga^3^^+^@αPD‐L1 treatment enhanced antigen‐specific CD8^+^ T cell activation (increased IFN‐γ and GZMB, Figure 4I) and elevated tumor cell death (PI^+^ cells, Figure 4J). Crucially, this enhanced cytotoxicity was accompanied by elevated Fe^2^^+^ (FerroOrange) and lipid peroxidation (Liperfluo) levels in B16‐OVA cells (Figure 4K,L). The specific involvement of ferroptosis was confirmed by rescue experiments, pre‐treatment with the inhibitors Lip‐1 or DFO effectively attenuated the increases in tumor cell death, lipid peroxidation, and Fe^2^^+^ accumulation (Figure 4J−L). Analysis of B16‐OVA cells from this co‐culture system demonstrated that *Lr*@TA/Ga^3^^+^@αPD‐L1 induced a characteristic ferroptosis‐associated expression profile: upregulation of ACSL4 alongside downregulation of GPX4, SLC7A11, and FSP1, which was reversed upon co‐administration of Lip‐1 or DFO (Figure 4M,N; Figure S2). In summary, *Lr*@TA/Ga^3+^@αPD‐L1 jointly induces tumor ferroptosis by concurrently disrupting iron metabolism via Ga^3^^+^ and activating antitumor immunity through PD‐L1 blockade.

### 2.4 Bio‐Distribution of *Lr*@TA/Ga^3+^@αPD‐L1 In Vivo

**FIGURE 5:**
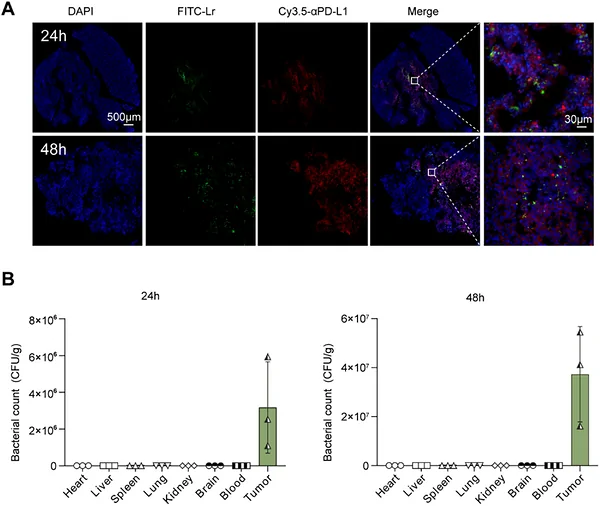
Biodistribution and organ colonization of *Lr*@TA/Ga^3+^@αPD‐L1. (A) Confocal images of tumor tissue 24 or 48 h after a single dose of intratumorally injected *Lr*@TA/Ga^3+^@αPD‐L1. The cell nucleus was stained with DAPI; *L. reuteri* was labeled with FITC; αPD‐L1 was labeled with Cy3.5. Scale bar: 500 and 30 µm. (B) Colony counts in major organs and tumors.

To further investigate the in vivo behavior of the constructed functionalized bacteria, LLC tumor‐bearing mouse model was established to assess the bio‐distribution of *Lr*@TA/Ga^3^^+^@αPD‐L1. The spatiotemporal distribution of *Lr*@TA/Ga^3^^+^@αPD‐L1 within tumor tissues was analyzed. Mice were administered a single intratumoral injection of *Lr*@TA/Ga^3^^+^@αPD‐L1 (2 × 10^5^ CFU) and euthanized at either 24 or 48 h post‐injection. Following excision, tumor tissues underwent digital fluorescence imaging analysis (fluorescence scanning system) to map the intratumoral distribution patterns of FITC‐conjugated *L. reuteri* and Cy3.5‐tagged αPD‐L1. At 24 h post‐injection, both fluorescent signals remained localized near the injection site (Figure 5A). By contrast, 48 h analysis revealed extensive dissemination of bacteria (FITC) and therapeutic antibody (Cy3.5) signals across tumor sections (Figure 5A), suggesting bacterial motility‐driven spatial redistribution of αPD‐L1 cargos.

Systemic bacterial distribution was quantitatively analyzed by CFU enumeration in tumor and major organ compartments. Per standardized protocols, cardiac, hepatic, splenic, pulmonary, renal, and tumor tissues were aseptically collected, homogenized, serially diluted, and cultured on selective De Man, Rogosa, and Sharp (MRS) agar under anaerobic conditions. Bacterial viable counts revealed marked tumor‐specific colonization, with bacterial loads exceeding 1 × 10^6^ CFU/g in neoplastic tissues, contrasting with minimal extra‐tumoral dissemination (Figure 5B; Figure S1B). This colonization and amplification capacity reflects adaptability of *L. reuteri* to the hypoxic, nutrient‐enriched, and mildly acidic tumor microenvironment, which collectively promote bacterial survival and evasion of immune‐mediated clearance mechanisms in normoxic tissues.

### 2.5 *Lr*@TA/Ga^3+^@αPD‐L1 Therapy has the Potential to Reprogram the Tumor Immune Microenvironment

**FIGURE 6:**
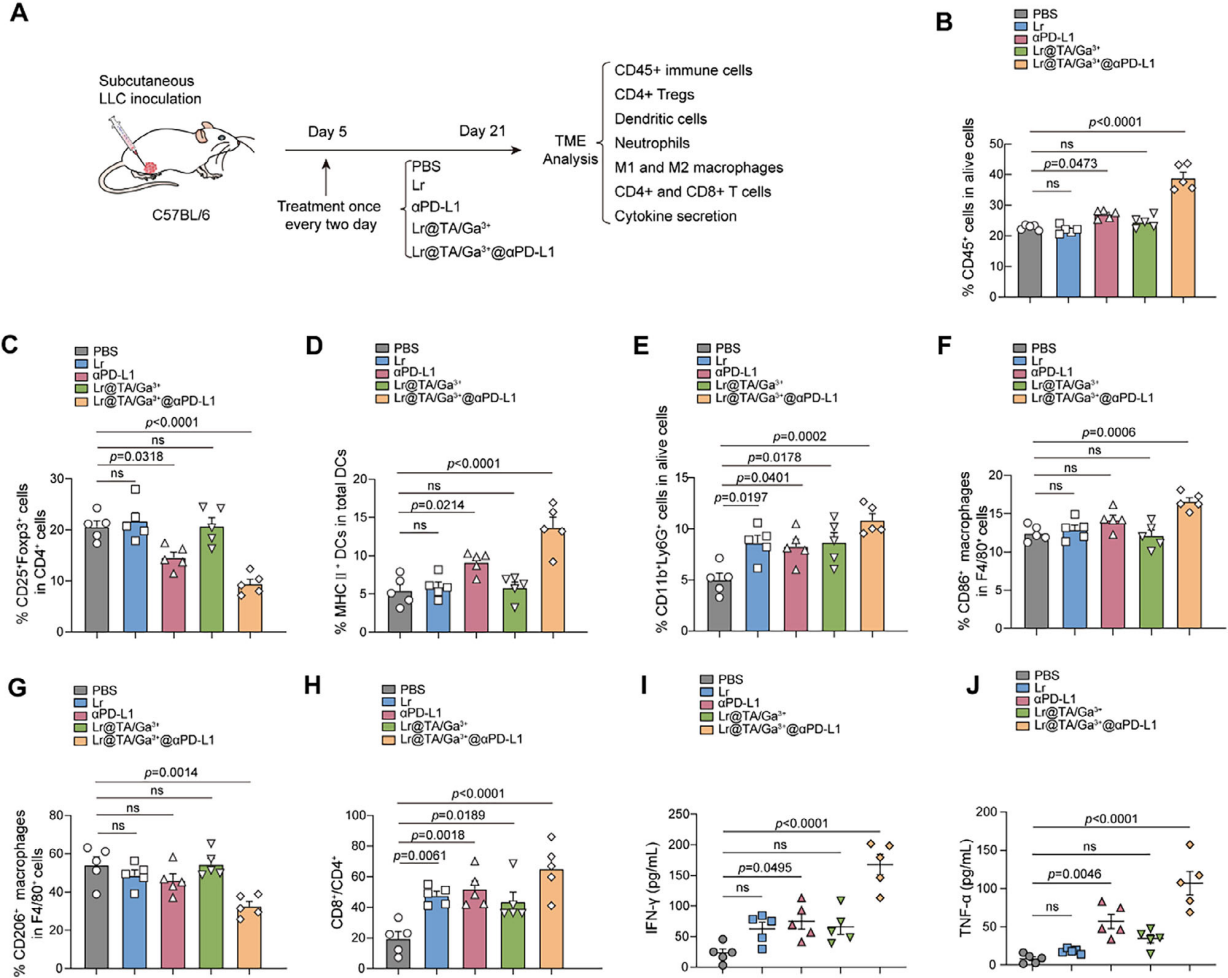
Intratumoral immune responses. (A) Experimental design of in vivo treatment in LLC tumor‐bearing mice. 1 × 10^6^ tumor cells were subcutaneously innoculated and 50 µL of blank PBS or PBS containing *Lr* (9 × 10^6^ CFU/mL), αPD‐L1 (1 mg/mL), *Lr*@TA/Ga^3+^ (9 × 10^6^ CFU/mL), or *Lr*@TA/Ga^3+^@αPD‐L1 (9 × 10^6^ CFU/mL) was intratumorally injected when the tumor size had reached 200 mm^3^. (B‐G) Immune responses in tumor sampled from mice with indicated treatments. Flow cytometric analysis of percentage of total immune cell (CD45^+^, B), Treg cells (CD25^+^Foxp3^+^, C), mature dendritic cells (CD11c^+^MHC II^+^, D), Neutrophil (CD11b^+^Ly6G^+^, E), and M1‐type to M2‐type macrophages (F and G). (H) Flow cytometric analysis of ratio of CD4^+^/CD8^+^ in CD3^+^ T cells. (I and J) Levels of IFN‐γ and TNF‐α in TIF measured by enzyme‐linked immunosorbent assay (ELISA). Statistical analysis was performed using one‐way ANOVA with the Tukey's post‐test (*n* = 5).

Commensal bacteria within the TME may modulate immune responses through various routes. To assess the immune response elicited by *Lr*@TA/Ga^3+^@αPD‐L1, a subcutaneous LLC tumor model was developed, and mice were intratumorally injected with phosphate‐buffered saline (PBS), *Lr*, αPD‐L1, *Lr*@TA/Ga^3+^, or *Lr*@TA/Ga^3+^@αPD‐L1, respectively. After 16 days of treatment, tumor tissues from treated mice were processed into single‐cell suspensions and analyzed via flow cytometry. The gating strategy for tumor‐infiltrating immune cells is indicated in Figure S3A,B. CD45^+^ immune cells, CD4^+^ and CD8^+^ T cells, dendritic cells, neutrophils, as well as M1 and M2 macrophages were included in the analysis (Figure 6A). We found that treatment with unmodified *Lr* significantly increased the CD8^+^/CD4^+^ T cell ratio and enhanced neutrophil infiltration, while not markedly affecting other immune cell types (Figure 6B‐J). Critically, the MPN coating (*Lr*@TA/Ga^3^^+^) preserved these inherent immunostimulatory properties of *Lr*, confirming that the coating itself does not compromise bacterial immunogenicity (Figure 6B‐J). By contrast, a significant increase in the infiltration of CD45^+^ immune cells in tumor tissues treated with *Lr*@TA/Ga^3+^@αPD‐L1 was observed, compared to all other groups (Figure 6B), indicating a robust immune response facilitated by the engineered bacterial system. Regulatory T cells (Tregs, CD25^+^Foxp3^+^) infiltration was decreased in the *Lr*@TA/Ga^3+^@αPD‐L1 treated group, indicating a reduction in immunosuppressive cell populations within the TME (Figure 6C). Also, mature dendritic cells (DCs, CD11c^+^MHC II^+^) in tumors were obviously up‐regulated after *Lr*@TA/Ga^3+^@αPD‐L1 treatment, indicating enhanced antigen presentation capacity (Figure 6D). Meanwhile, neutrophils (CD11b^+^Ly6G^+^) were increased in the *Lr*@TA/Ga^3+^@αPD‐L1 treatment group (Figure 6E). The ratio of M1‐type (CD86^+^) to M2‐type (CD206^+^) macrophages increased in the *Lr*@TA/Ga^3+^@αPD‐L1 group, indicating a shift toward a pro‐inflammatory macrophage phenotype (Figure 6F,G). Notably, *Lr*@TA/Ga^3+^@αPD‐L1 treatment resulted in the highest ratio of CD8^+^/CD4^+^ T cell ratio in lymphocytes (Figure 6H) and the highest secretion amount of IFN‐γ and tumor necrosis factor‐α (TNF‐α) in tumor interstitial fluid (TIF) among all treated groups (Figure 6I,J), demonstrating the elicitation of a strong cytotoxic T cell response inside the tumor. Taken together, the native *L. reuteri* strain possesses immunomodulatory activity, the MPN coating strategy successfully retains this function, and *Lr*@TA/Ga^3+^@αPD‐L1 can remodel the immunosuppressive TME and elicit a potent antitumor immune response by significantly increasing the infiltration of CD45^+^ immune cells, cytotoxic T cells, and mature DCs, reducing the proportion of Tregs, polarizing macrophages toward the M1 phenotype, and promoting the secretion of IFN‐γ and TNF‐α.

### 2.6 Synergistically Enhanced Immunotherapy in Tumor Models In Vivo

**FIGURE 7:**
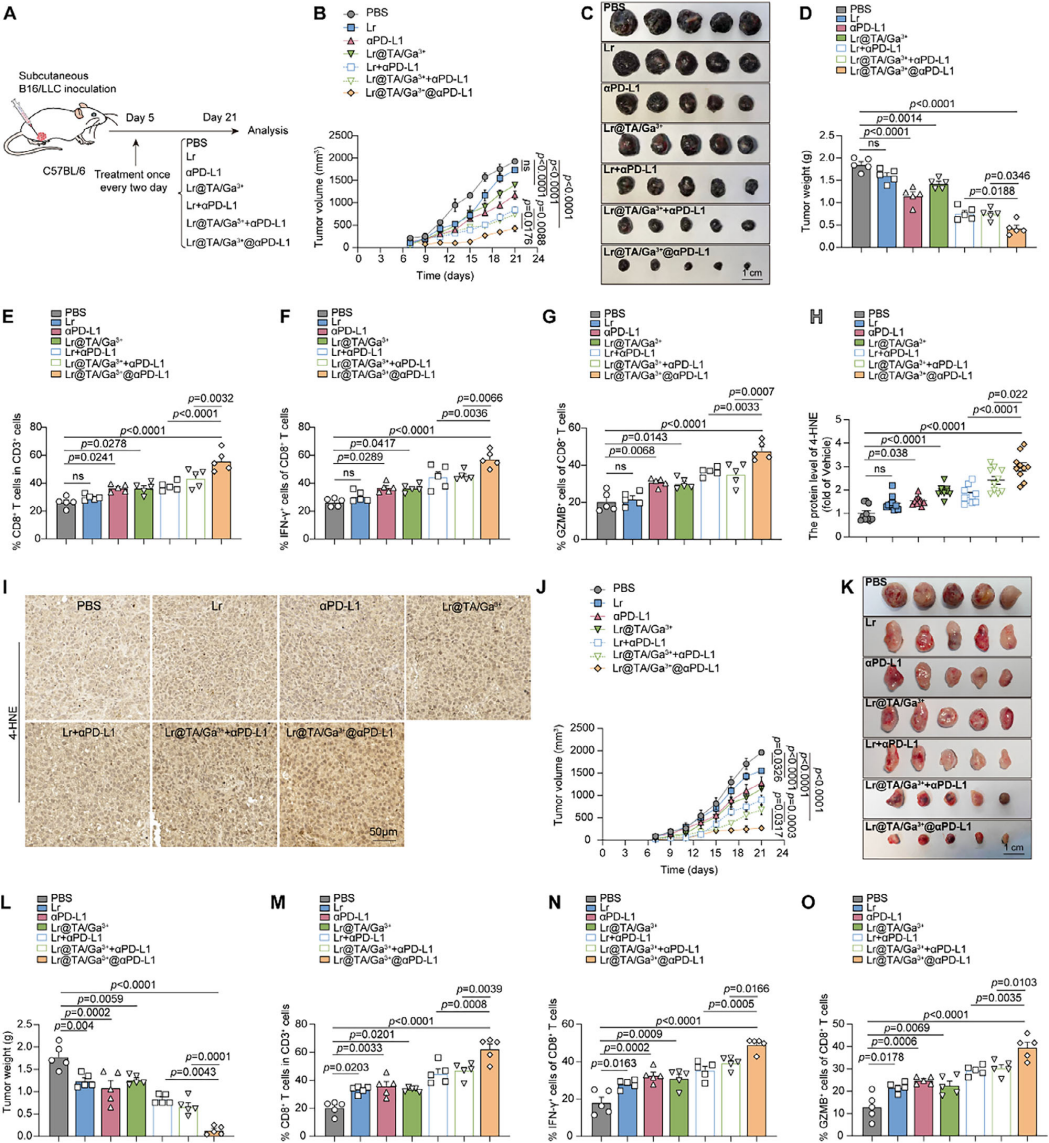
Antitumor responses generated by *Lr*@TA/Ga^3+^@αPD‐L1. (A) The strategy for investigating the antitumor effects of 50 µL blank PBS or PBS containing *L. reuteri* (9 × 10^6^ CFU/mL), αPD‐L1 (1 mg/mL), *Lr*@TA/Ga^3+^ (9 × 10^6^ CFU/mL), *Lr*@TA/Ga^3+^@αPD‐L1 (9 × 10^6^ CFU/mL), *Lr* (9 × 10^6^ CFU/mL) *+* αPD‐L1 (50 µg/injection), or *Lr*@TA/Ga^3+^ (9 × 10^6^ CFU/mL) *+* αPD‐L1 (50 µg/injection) treatment in vivo. (B‐D) Tumor volume (B), representative images (C) and tumor weight (D) of LLC tumor‐bearing mice with indicated treatments. Scale bar, 1 cm. (E–G) The percentage of CD8^+^ T cells in CD3^+^ tumor infiltrating T cells (E), as well as IFN‐γ and GZMB expression of tumor infiltrating CD8^+^ T cells (F and G). (H and I) The IHC staining and quantitation of 4‐HNE expression in LLC tumor tissue. Scale bar, 50 µm. (J–N) Effects of the indicated treatments on B16 tumor growth (J), representative images (K), tumor weight (L), the percentage of CD8^+^ T cells in CD3^+^ tumor infiltrating T cells (M), as well as IFN‐γ and GZMB expression of tumor infiltrating CD8^+^ T cells (N and O). Scale bar, 1 cm. Statistical analysis was performed using one‐way ANOVA with the Tukey's post‐test (*n* = 5).

To evaluate the antitumor activity of functionalized bacteria in vivo, we examined the therapeutic effects of *Lr*@TA/Ga^3+^@αPD‐L1 in tumor growth inhibition in LLC and B16 tumor‐bearing mice, respectively. The subcutaneous cancer models were established by injecting LLC or B16 cells into the right flank of mice subcutaneously. Five days after tumor inoculation, 5×10^6^ CFU *Lr*@TA/Ga^3+^@αPD‐L1 were dosed as prime immunization by intratumoral injection. PBS and equivalent αPD‐L1, *Lr*, *Lr*@TA/Ga^3+^, the non‐coassembled mixture treatment *Lr* + αPD‐L1, or *Lr*@TA/Ga^3^^+^ + αPD‐L1 were set as controls, respectively (Figure 7A). Compared with the control groups, *Lr*@TA/Ga^3+^@αPD‐L1 treatment significantly delayed tumor progression, as evidenced by the tumor volume measured every other day and the final tumor weight (Figure 7B−D). *Lr*@TA/Ga^3+^@αPD‐L1 treatment increased the number of CD8^+^ T cells infiltration in tumor (Figure 7E) and concurrently upregulated the expression of the effector molecules GZMB and IFN‐γ of CD8^+^ T cells in vivo (Figure 7F,G). Furthermore, the immunohistochemical analysis revealed *Lr*@TA/Ga^3+^@αPD‐L1 group induced a significant upregulation of 4‐hydroxynonenal (4‐HNE), which is an end product of ferroptosis (Figure 7H,I). Similar results were obtained in B16 tumor model, treatment with *Lr*@TA/Ga^3+^@αPD‐L1 further attenuated B16 tumor growth compared with the effect of either control treatment (Figure 7J−L). CD8^+^ TIL infiltration and effector function were also restored after administration of *Lr*@TA/Ga^3+^@αPD‐L1 (Figure 7M−O). Critically, the therapeutic and immunostimulatory outcomes achieved with the co‐assembled *Lr*@TA/Ga^3^^+^@αPD‐L1 platform were significantly greater than those observed with the simple mixtures (*Lr* + αPD‐L1 and *Lr*@TA/Ga^3^^+^ + αPD‐L1), supporting a synergistic rather than merely additive mechanism. These findings suggested that therapy consisting of *Lr*@TA/Ga^3+^@αPD‐L1 shows potential benefit for tumor immunotherapy.

## 3 Discussion

The anti‐tumor efficacy of the *Lr*@TA/Ga^3^^+^@αPD‐L1 platform could sequentially disrupt key tumor survival pathways while simultaneously activating and sustaining a potent immune response. The initial and critical step is the hypoxia‐driven tropism of the functionalized *L. reuteri*. Solid tumors' characteristic dysregulated vasculature creates pronounced hypoxic and necrotic regions. Facultative anaerobes like *L. reuteri* inherently migrate toward and colonize these oxygen‐depleted niches, achieving high intratumoral enrichment. This biological targeting ensures a high local concentration of therapeutic agents directly within the immunosuppressive tumor core.

Upon colonization, the acidic tumor microenvironment (pH 6.5–6.8) triggers the pH‐responsive disassembly of the MPN coating. The coordination bonds between TA and Ga^3^^+^ weaken under acidic conditions, leading to the release of both cargoes. This ensures that αPD‐L1 antibodies and Ga^3^^+^ are predominantly liberated within the tumor, minimizing off‐target effects and maximizing local drug accumulation. The released αPD‐L1 antibodies execute immune checkpoint blockade by interrupting the PD‐1/PD‐L1 inhibitory axis. This “releases the brakes” on pre‐existing tumor‐infiltrating lymphocytes (TILs), particularly CD8^+^ T cells, restoring their cytotoxic effector functions such as IFN‐γ and granzyme B secretion.

Concurrently, the released Ga^3^^+^ ions initiate a dual‐pronged attack on tumor iron metabolism to induce ferroptosis. Structurally mimicking Fe^3^^+^, Ga^3^^+^ competes with Fe^3^^+^ for binding to transferrin, disrupting iron uptake and cellular iron homeostasis. Notably, the ferroptotic death of tumor cells is intrinsically immunogenic. The release of damage‐associated molecular patterns (DAMPs), such as lipid peroxidation products (e.g., 4‐HNE) and high‐mobility group box 1 (HMGB1), acts as an “eat‐me” signal, promoting the phagocytosis of dead tumor cells by DCs. This process enhances DC maturation and cross‐presentation of tumor antigens, thereby priming and recruiting *de novo* tumor‐specific T cells—a process effectively turning the tumor “hot”. Crucially, these pathways are not parallel but form a self‐reinforcing positive feedback loop. The Immune checkpoint blockade (ICB)‐reactivated CD8^+^ T cells secrete IFN‐γ, which further amplifies ferroptosis.

Chemically engineered bacteria hold significant promise for future clinical applications, including the development of precision therapies such as targeted drug delivery systems, intelligent diagnostics, and living therapeutics for modulating the gut microbiome or treating metabolic disorders [24, 25]. However, their clinical translation faces several key challenges. These obstacles include ensuring long‐term biosafety and genetic stability, achieving precise spatial and temporal control of bacterial activity within the human body [26], managing potential immune responses, navigating complex regulatory pathways, and developing scalable and cost‐effective manufacturing processes. Addressing these hurdles is crucial for realizing the full therapeutic potential of this innovative technology.

## 4 Conclusion

Herein, we report an engineered bacterial‐based material that integrates MPNs and ICIs to stimulate antitumor immunity and induce ferroptosis, thereby achieving multimodal tumor eradication [27]. In vivo evaluations in B16 and LLC tumor‐bearing mouse models reveal that L. reuteri functionalized with αPD‐L1 antibodies and Ga^3^^+^‐MPNs significantly suppressed tumor growth. This multi‐modification strategy provides a versatile platform for developing potent combinatorial immunotherapies. By integrating bacterial tropism, MPN‐mediated ferroptosis induction, and ICI‐driven immune activation, this approach addresses key limitations of monotherapies, offering new avenues for treating refractory solid tumors and immunologically “cold” malignancies. The work highlights the potential of chemical engineered bacteria as versatile carriers for next‐generation combinatorial immunotherapy.

## 5 Experimental Section

### 5.1 Animal Studies

C57BL/6 mice (6−7 weeks old, male) were purchased from Beijing Hua Fu Kang Bioscience Co., Ltd. (Beijing, China). All mice were maintained in the animal facility at the Institute of Medicinal Biotechnology (approval number: SYXK 2022‐0023) under specific‐pathogen‐free (SPF) conditions. For the animal studies, the mice were earmarked before grouping and then randomly separated into groups by an independent person; however, no particular randomization method was used. The sample size was predetermined empirically according to previous experience using the same strains and treatments. Generally, we used n ≥ 6 mice per genotype and condition. All animal procedures were conducted in accordance with the guidelines of the Institutional Committee for the Ethics of Animal Care and Treatment in Biomedical Research of the Chinese Academy of Medical Sciences and Peking Union Medical College (Permit No. IMB‐20221228D501). The animal study was also performed in accordance with the ARRIVE guidelines.

### 5.2 Cell Lines

B16 and LLC tumor cells were purchased from Cell Resource Center, Peking Union Medical College (the headquarter of National Science & Technology Infrastructure—National BioMedical Cell‐Line Resource, NSTI‐BMC) (September, 2022, Beijing, China). LLC cells were cultured and maintained in RPMI‐1640 medium (Thermo Fisher Scientific, cat. 11875500) supplemented with 10% FBS (Thermo Fisher Scientific, cat. 10091–148) under 5% carbon dioxide (CO_2_). B16 cells were cultured and maintained in DMEM medium (Thermo Fisher Scientific, cat. 11995500) supplemented with 10% FBS under 5% CO_2_. All cell lines were verified negative for mycoplasma contamination by One‐Step Mycoplasma Detection Kit (Yeasen Biotechnology, cat. 40612ES25). No cells lines used here appear in the database of commonly misidentified cell lines (the International Cell Line Authentication Committee).

### 5.3 Construction and Characterization of Functionalized Materials

L. reuteri DSM17938 (Hangzhou Baosai Biotech, cat. E2027) were cultured overnight in MRS medium (hopebiol, cat. HB0384‐1), followed by a single wash with PBS to remove residual medium. The bacterial pellets were resuspended in Bicine buffer (100 mM, pH 8.5, Aladdin, cat. B110906) containing 100 µM TA (Innochem, cat. A38870) and 60 µM Ga(NO_3_)_3_ (Innochem, cat. A84204) and incubated at room temperature for 3 h under gentle agitation. Unbound TA and Ga(NO_3_)_3_ were removed by centrifugation (3 000 rpm, 5 min) to obtain Lr@TA/Ga^3+^. Subsequently, the obtained bacteria were further incubated with 200 µg of PD‐L1 antibody (BioXcell, cat. BE0101, RRID: AB_10949073) for 3 h. Unconjugated antibodies were removed by centrifugation (3 000 rpm, 5 min), yielding the dual‐functionalized Lr@TA/Ga^3+^@αPD‐L1. The final product was washed three times with PBS and stored at 4°C for subsequent use.

The morphological features of the functionalized bacteria were characterized by TEM employing negative staining techniques. The structural assembly of the constructs was confirmed by confocal laser scanning microscopy (CLSM), with L. reuteri fluorescently labeled with FITC (Solarbio, cat. IF0081) and αPD‐L1 conjugated to Cy3.5(DuoFluor, cat. D10011‐5) for multimodal imaging. Size distribution profiles and surface charge (zeta potential) were quantitatively assessed by DLS utilizing a nanoparticle size analyzer. UV‐Vis spectra of TA, Ga(NO_3_)_3_, Lr, Lr@TA/Ga^3+^, and Lr@TA/Ga^3+^@αPD‐L1 were recorded by Mettler Toledo UV5. FTIR spectra was recorded by Thermo scientific Nicolet Summit Lite.

The antibody loading capacity of the functionalized bacteria was determined using BCA assay (BCA Protein Assay Kit, Solarbio, cat. PC0020) by measuring the protein concentration in the initial reaction mixture and in the supernatant after reaction completion. To evaluate the release of the payloads, the functionalized bacteria was dispersed in PBS (pH 6.8 and 7.4) supplemented with 10% FBS and incubated at 37°C. Samples were collected at 0, 6, and 12 h, centrifuged, and resuspended in an equal volume of fresh PBS. Fluorescence intensity was measured at each time point to evaluate the release rate of the material under different pH conditions.

### 5.4 ICP‐MS Analysis

ICP‐MS was employed for gallium content determination. LLC cells were cultured to 80% confluence. Anti‐CD71 (abinScience, cat. HY036096) was added at a concentration of 5 µg/mL and incubated for 1 h. Subsequently, Lr@TA/Ga^3^^+^@αPD‐L1 was added to the cells at a dose equivalent to 5 µg/mL PD‐L1 and incubated for 24 h. Cells were washed twice with PBS, collected, and the gallium content in the cells was detected by ICP‐MS.

### 5.5 Binding Capacity of Functionalized Bacteria to Tumor Cells

Binding capacities of Lr, Lr@TA/Ga^3^^+^, and Lr@TA/Ga^3^^+^@αPD‐L1 to tumor cells were evaluated using LLC cells. LLC cells (2 × 10^5^ cells/well) were seeded into 24‐well plates or glass‐bottom imaging dishes. After 12 h, 1 × 10^5^ CFU of Lr, Lr@TA/Ga^3^^+^, or Lr@TA/Ga^3^^+^@αPD‐L1 were added to the cultures and incubated for 3 h at 37°C. L. reuteri were pre‐labeled with FITC (Solarbio, cat. IF0080) for visualization. Cells were subsequently washed once with PBS to remove unbound bacteria and harvested for flow cytometric and confocal analysis. For confocal imaging, cells in glass‐bottom dishes were stained with Hoechst 33342 (Beyotime, cat. C1026) (nuclear dye) and DiD (Beyotime, cat. C19955) (membrane dye), followed by imaging using a laser scanning confocal microscope.

### 5.6 Mouse Tumor Growth Model

To establish the tumor growth model in C57BL/6 mice, 1 × 10^6^ LLC or 5 × 10^6^ B16 cells were subcutaneously injected into the right flanks of the mice. To evaluate the antitumor effects, tumor‐bearing C57BL/6 mice were treated with PBS, *Lr* (9 × 10^6^ CFU), αPD‐L1 (50 µg/injection, clone 10F.9G2, Bio X Cell, Cat: #BE0101, RRID AB_10949073), *Lr*@TA/Ga^3+^ (9 × 10^6^ CFU), *Lr*@TA/Ga^3+^@αPD‐L1 (9 × 10^6^ CFU, equivalent to 50 µg αPD‐L1), *Lr* (9 × 10^6^ CFU/mL) *+* αPD‐L1 (50 µg/injection), or *Lr*@TA/Ga^3+^ (9 × 10^6^ CFU/mL) *+* αPD‐L1 (50 µg/injection) via intratumoral injection (50 µL, every two days). Tumor volumes were measured by caliper every two days when palpable tumors were presented. Tumor volumes were calculated using the following formula of ellipsoid volume: V = D × d × d / 2, where D represents the longest diameter and represents the shortest diameter. Mice were euthanized at predetermined humane endpoints.

### 5.7 CD8^+^ T Cells Isolation and Culture

For mouse CD8^+^ T cells isolation, spleens and lymph nodes collected from C57BL/6 mice were processed into single‐cell suspension. CD8^+^ T cells were purified with the ImunoSep Mouse CD8^+^ T cell Isolation Kit (Precision BioMedicals Co., Ltd., Tianjin, China; cat. 720820) with immunomagnetic negative selection according to manufacturer's protocol. The purity of CD8^+^ T cells was confirmed by flow cytometry (>95%) using an anti‐CD8 antibody. The isolated CD8^+^ T cells were cultured in RPMI 1640 medium supplemented with 10% FBS, 10 ng/mL recombinant murine IL‐2 (Peprotech, cat. 212‐12) and 1% penicillin/streptomycin. CD8^+^ T cells were activated with anti‐CD3 antibody (2 µg/mL, Biogems, cat. 05112‐25‐500, RRID: AB_3099697) and anti‐CD28 antibody (2 µg/mL, Biogems, cat. 10312‐25‐599, RRID: AB_3099698) for 48 h or indicated time periods.

### 5.8 Flow Cytometry

To assess tumor cell death, tumor cells were pelleted and washed twice in PBS buffer, followed by staining with PI using Cellstain‐ PI solution (Dojindo, cat. P346) according to the instructions provided by the manufacturer.

For the analysis of surface markers, single‐cell suspensions of tumor infiltrating immune cells were prepared by mechanical dissociation, resuspended in MACS buffer (consisting of PBS with 2% FBS and 2 mM EDTA), and stained with the indicated antibodies. Antibodies: PerCP/Cy5.5 anti‐mouse CD3 (Biolegend, cat. 100218, RRID: AB_1595492); APC‐Cy7 anti‐mouse CD8 (Biolegend, cat. 100714, RRID: AB_312753); Alexa Flour 488 anti‐mouse CD8a (Biolegend, cat. 100726, RRID: AB_493423); PE anti‐mouse CD4 (Biolegend, cat. 100512, RRID: AB_312715); FITC anti‐mouse CD4 (Biolegend, cat. 100406, RRID: AB_312691); FITC anti‐mouse CD25 (Biolegend, cat. 101908, RRID: AB_961212); BUV496 anti‐mouse I‐A/I‐E (MHC II) (BD Biosciences, cat. 750281, RRID: AB_2874472); APC anti‐CD11c (Biolegend, cat. 117305, RRID: AB_313774); APC anti‐mouse CD45 (Biolegend, cat. 103108, RRID: AB_312973); APC anti‐mouse CD11b (eBioscience, cat. 47‐0112‐80, RRID: AB_1603195), PE anti‐mouse Ly6G (Biolegend, cat. 127607, RRID: AB_1186104); FITC anti‐mouse CD45 (Biolegend, cat. 103108, RRID: AB_312973); PE anti‐mouse F4/80 (Biolegend, cat. 111703, RRID: AB_2936728); and APC anti‐mouse CD86 (Biolegend, cat. 105007, RRID: AB_313150) were incubated for 1 h at room temperature and then cells were washed twice by MACS buffer. For intracellular staining, cells were stimulated with 30 nM Phorbol myristate acetate (PMA, Sigma Aldrich, cat. P1585), protein transport inhibitor (1:1000, BD Biosciences, cat. 554724) and 1 µM ionomycin (Sigma Aldrich, cat. I3909) supplemented with 2 µg/mL brefeldin A (Selleck, cat. S7046) for 4−6 h at 37°C. Following surface staining, the cells were fixed with fixation buffer (BD, cat. 554655), and permeabilized with 1 × permeabilization buffer (BD, cat. 554723) for 30 min at room temperature. The following fluorescently labeled antibodies were used for intracellular staining: PerCP/Cy5.5 anti‐mouse/human GZMB (Biolegend, cat. 372212, RRID: AB_2728379); PE‐Cy7 anti‐mouse IFN‐γ (Biolegend, cat. 505826, RRID: AB_2295770); Alexa Flour 488 anti‐mouse FOXP3 (Biolegend, cat. 320012, RRID: AB_439748); and PE/Cy7 anti‐mouse CD206 (Biolegend, cat. 141719, RRID: AB_2562247). Data were acquired using CytoFLEX Flow Cytometer (Beckman) and analyzed by FlowJo 10.8.1 software.

### 5.9 In Vitro Cell‐Killing Assay

The isolated CD8^+^ T cells were preactivated by anti‐CD3/anti‐CD28 antibodies or OVA peptide for 48 h. For the in vitro tumor cell‐killing assay, 3 × 10^5^ LLC cells or B16‐F10 OVA cells were plated on 12‐well plates and incubated at 37°C for 24 h for attachment. Subsequently, the preactivated CD8^+^ T cells were plated on the upper medium of LLC cells. CD8^+^ T cells isolated from C57BL/6 mice were cocultured with LLC tumor cells at a ratio of 5:1 (CD8^+^ T cells: tumor cells) supplemented with IL‐2 for 36 h. CD8^+^ T cells isolated from OT‐1 mice were cocultured with B16‐F10 OVA tumor cells at a ratio of 3:1 (CD8^+^ T cells: tumor cells) supplemented with IL‐2 for 48 h. After coculture, cells were detached with trypsin, and resuspended in MACS buffer. The proportion of dead tumor cells was determined by flow cytometry to analyze the tumor cell‐killing effectiveness of the CD8^+^ T cells. For the analysis of GZMB and IFN‐γ, single‐cell suspensions of CD8^+^ T cells were prepared, resuspended in MACS buffer, stained with the indicated antibodies, and analyzed by flow cytometry.

### 5.10 Lipid Peroxidation Detection with LiperFluo

Following the co‐culture period, LLC and B16‐OVA cells were processed for lipid peroxidation assessment using the LiperFluo probe (Dojindo). Briefly, the culture medium was removed, and the cells were gently washed once with serum‐free medium. Subsequently, 200 µL of serum‐free RPMI‐1640 medium containing 1 µM LiperFluo was added to cover the cells. The cells were then incubated in the dark at 37°C under 5% CO_2_ for 30 min. After incubation, the staining solution was aspirated, and the cells were washed twice with 200 µL of Hanks' Balanced Salt Solution (HBSS). Finally, the fluorescence intensity of the tumor cell population was analyzed immediately by flow cytometry using 488 nm excitation and a 500–550 nm emission filter set.

### 5.11 Divalent Iron Ion Detection with FerroOrange

Tumor cells were stained with the Fe^2^^+^‐specific fluorescent probe FerroOrange (Dojindo) to visualize the intracellular Fe^2^^+^ pool. LLC or B16‐OVA cells were seeded into glass‐bottom confocal dishes and allowed to adhere overnight in a 37°C, 5% CO_2_ incubator. After the designated co‐culture period, the medium was aspirated and the cells were gently washed once with serum‐free medium. Subsequently, 200 µL of a 1 µM FerroOrange working solution, prepared in serum‐free RPMI‐1640 medium, was added to the cells. The dishes were then incubated in the dark at 37°C under 5% CO_2_ for 30 min. Following incubation, the staining solution was removed, and the cells were washed twice with 200 µL of Hanks' Balanced Salt Solution (HBSS). The fluorescence signal from the tumor cells was immediately captured using a confocal fluorescence microscope with appropriate filter sets for FerroOrange detection.

### 5.12 PD‐1/PD‐L1 Binding Blockade Assay

PD‐L1‐expressing LLC tumor cells were harvested and seeded into a 96‐well plate. The cells were first incubated with a recombinant mouse PD‐1/Fc chimera protein (2 µg/mL) for 1 h at 4°C to allow binding to cell surface PD‐L1. Following this, the cells were treated with PBS (negative control), free αPD‐L1 antibody (positive control), or Lr@TA/Ga^3^^+^@αPD‐L1 for an additional 1 h at 4°C. Unbound reagents were then removed by washing with cold FACS buffer. To detect the level of PD‐1/Fc chimera remaining bound to PD‐L1, the cells were stained with a PE‐conjugated anti‐mouse IgG Fc secondary antibody for 30 min at 4°C in the dark. Finally, the cells were washed, resuspended, and analyzed by flow cytometry. The mean fluorescence intensity (MFI) of PE was measured, and the percentage of binding inhibition was calculated relative to the PBS‐treated control.

### 5.13 Immunohistochemical Analysis

Tissue sections were deparaffinized in xylene and rehydrated through a graded alcohol series and distilled water. Antigen retrieval was carried out in citrate buffer (10 mM sodium citrate buffer, pH 6.0) at a sub‐boiling temperature for 15 min in a microwave. Sections were permeabilized with 0.5% Triton X‐100 in PBS for 20 min. Endogenous peroxidase activity was blocked with 3% H_2_O_2_ solution for 10 min, after which the sections were then washed three times with PBS. Blocking buffer (3% bull serum albumin/PBS) was added to the sections and incubated for 30 min. The sections were then incubated with the indicated primary antibodies (4‐Hydroxynonenal Monoclonal antibody, Proteintech, cat. 68538‐1‐Ig, RRID: AB_3085246) at 4°C overnight. After washing three times, the sections were incubated for 30 min with the corresponding secondary antibodies at room temperature. Signals were stained with freshly prepared DAB substrate solution (ZSGB‐BIO Company, Beijing, China). The sections were then counterstained with hematoxylin, dehydrated, and mounted with coverslips. Images were acquired using an Olympus DP72 microscope (Olympus Microsystems, CA, USA) and analyzed with Image‐Pro Plus 5.1 software.

### 5.14 Enzyme‐Linked Immunosorbent Assay (ELISA)

The expression levels of mouse GZMB and IFN‐γin TIF was determined using ELISA kit (Beijing 4A Biotech Co., Ltd., China) according to the manufacturer's instructions. Tumor tissues were centrifuged against a 70 µm cell strainer at 4°C for 5 min at 500 g. Then the flow‐through TIF was centrifugated at 4°C for 5 min at 500 g. Supernatant were flash‐frozen and stored at −80°C until analysis. The GZMB and IFN‐γ levels in each group of TIF were quantified. Plates were scanned at 450 nm using Synergy H1 microplate reader (BioTek). The concentrations of cytokines in the unknown samples were then determined by comparing the optical density of the samples to the standard curve. Independent experiments were performed in triplicate.

### 5.15 Quantification and Statistical Analysis

Analyses were performed using GraphPad Prism 8.0 software with significance set to *p* < 0.05. The numbers of samples and independent biological experimental repeats are indicated in the figures or figure legends. All quantitative data are expressed as means ± SEMs. Statistical analysis was performed using one‐way analysis of variance (ANOVA) with the Tukey's post‐test.

## Author Contributions

T.Z. completed animal model construction and flow cytometry analysis; M.X. and Y.Z. completed material preparation and characterization; Z.L. and D.S. performed method optimization; J.P. drafted the manuscript; K.L. and Y.W. revised and reviewed the manuscript.

## Conflicts of Interest

The authors declare no conflicts of interest.

## Supporting information




**Supporting File**: advs74935‐sup‐0001‐SuppMat.docx

## Data Availability

The data that support the findings of this study are available from the corresponding author upon reasonable request.
