# Supplementary material for: Chemically Engineered L. reuteri Delivering αPD‐L1 and Gallium Ions via Metal‐Phenolic Networks Potentiate Anti‐Tumor Immunity and Ferroptosis
Source: Adv Sci (Weinh). 2026 Mar 20;13(31):e21638. doi: 10.1002/advs.202521638 (PMC13588008; doi:10.1002/advs.202521638)
Supplement: Supplementary file 1 [file ADVS-13-e21638-s001.docx]

Chemically engineered *L. reuteri* Delivering αPD-L1 and Gallium Ions *via* Metal-Phenolic Networks Potentiate Anti-Tumor Immunity and Ferroptosis

Tingting Zhang^†,a^, Mengyi Xu^†,a,b^, Yan Zhang^a^, Zhiwen Li^b^, Danqing Song^a^, Jing Pang*^,a^, Ke Li*^,a^, Yanxiang Wang*^,b^

*^a^Institute of Medicinal Biotechnology,* *Chinese Academy of Medical Sciences and Peking Union Medical College, Beijing 100050, China*

*^b^Institute of Health and Medicine, Hefei Comprehensive National Science Center, Hefei, 230601, Anhui, China*

Correspondence: Jing Pang (pangjing@imb.pumc.edu.cn), Ke Li (like1986@163.com), Yanxiang Wang (wangyanxiang@ihm.ac.cn)

^†^ These authors contributed equally: Tingting Zhang, Mengyi Xu

**Figure S1**. (A) Bacterial plate counts of modified bacteria and bare bacteria; (B) Intratumoral colony count 48 hours after administration.

**Figure S2**. mRNA level of GPX4 and FSP1 in CD8^+^ T cell isolated from C57 (A) and OT-I transgenic mice (B).

**Figure S3**. Gating strategies for flow cytometric analysis. (A and B) Flow cytometry gating strategy for immune cells and CD8^+^ T cells for the intracellular expression of IFN-γ, GZMB and Ki67 in LLC tumor.

**Table S1**. Cytotoxicity Assay

| Name | CC_50_ | Name | CC_50_ |
| --- | --- | --- | --- |
| *Lr* | > 2×10^5^CFU | TA | 102 μM |
| *Lr*@TA/Ga^3+^ | > 2×10^5^CFU | Ga(NO_3_)_3_ | 360.9 μM |
| *Lr*@TA/Ga^3+^@αPD-L1 | > 2×10^5^CFU |  |  |

**Table S2**. Reagents used in this study

| Name | Manufacturers | CAS |
| --- | --- | --- |
| Tannic acid | A38870, Innochem | 1401-55-4 |
| Galllum nitrate hydrate | A84204, Innochem | 69365-72-6 |
| FITC | IF0081, Solarbio | 3326-32-7 |
| Cy3.5 | D10011-5, DuoFluor | 2231670-85-0 |
| Bicine | B110906, Aladdin | 150-25-4 |

**Table S3**. Antibody used in this study

| Name | Manufacturers | Application | RRIDs |
| --- | --- | --- | --- |
| anti-mouse PD-L1 | BE0101, BioXcell | *In vivo* and *in vitro* PD-L1 blockade | AB_10949073 |
| anti-CD3 | 05112-25-500, Biogems | CD8^+^ T cells activate | AB_3099697 |
| anti-CD28 | 10312-25-599, Biogems | CD8^+^ T cells activate | AB_3099698 |
| PerCP/Cy5.5 anti-mouse CD3 | 100218, Biolegend | Flow cytometry | AB_1595492 |
| APC-Cy7 anti-mouse CD8 | 100714, Biolegend | Flow cytometry | AB_312753 |
| Alexa Flour 488 anti-mouse CD8a | 100726, Biolegend | Flow cytometry | AB_493423 |
| PE anti-mouse CD4 | 100512, Biolegend | Flow cytometry | AB_312715 |
| FITC anti-mouse CD4 | 100406, Biolegend | Flow cytometry | AB_312691 |
| FITC anti-mouse CD25 | 101908, Biolegend | Flow cytometry | AB_961212 |
| BUV496 anti-mouse I-A/I-E (MHC II) | 750281, BD Biosciences | Flow cytometry | AB_2874472 |
| APC anti-CD11c | 117305, Biolegend | Flow cytometry | AB_313774 |
| APC anti-mouse CD45 | 103108, Biolegend | Flow cytometry | AB_312973 |
| APC anti-mouse CD11b | 47-0112-80, eBioscience | Flow cytometry | AB_1603195 |
| PE anti-mouse Ly6G | 127607, Biolegend | Flow cytometry | AB_1186104 |
| FITC anti-mouse CD45 | 103108, Biolegend | Flow cytometry | AB_312973 |
| PE anti-mouse F4/80 | 111703, Biolegend | Flow cytometry | AB_2936728 |
| APC anti-mouse CD86 | 105007, Biolegend | Flow cytometry | AB_313150 |
| PerCP/Cy5.5 anti-mouse/human GZMB | 372212, Biolegend | Flow cytometry | AB_2728379 |
| PE-Cy7 anti-mouse IFN-γ | 505826, Biolegend | Flow cytometry | AB_2295770 |
| Alexa Flour 488 anti-mouse FOXP3 | 320012, Biolegend | Flow cytometry | AB_439748 |
| PE/Cy7 anti-mouse CD206 | 141719, Biolegend | Flow cytometry | AB_2562247 |
| 4-Hydroxynonenal Monoclonal antibody | 68538-1-Ig, Proteintech | Immunohistochemical analysis | AB_3085246 |
